# Characterization of core fucosylation via sequential enzymatic treatments of intact glycopeptides and mass spectrometry analysis

**DOI:** 10.1038/s41467-022-31472-4

**Published:** 2022-07-07

**Authors:** Liwei Cao, T. Mamie Lih, Yingwei Hu, Michael Schnaubelt, Shao-Yung Chen, Yangying Zhou, Chuanyu Guo, Mingming Dong, Weiming Yang, Rodrigo Vargas Eguez, Lijun Chen, David J. Clark, Akrit Sodhi, Qing Kay Li, Hui Zhang

**Affiliations:** 1 Department of Pathology, Johns Hopkins University, Baltimore, MD 21231 USA; 2 Wilmer Eye Institute, Johns Hopkins University School of Medicine, Baltimore, MD 21287 USA

**Keywords:** Proteomics, Glycosylation, Glycoproteins, Mass spectrometry

## Abstract

Core fucosylation of N-linked glycoproteins has been linked to the functions of glycoproteins in physiological and pathological processes. However, quantitative characterization of core fucosylation remains challenging due to the complexity and heterogeneity of N-linked glycosylation. Here we report a mass spectrometry-based method that employs sequential treatment of intact glycopeptides with enzymes (STAGE) to analyze site-specific core fucosylation of glycoproteins. The STAGE method utilizes Endo F3 followed by PNGase F treatment to generate mass signatures for glycosites that are formerly modified by core fucosylated N-linked glycans. We benchmark the STAGE method and use it to characterize site specific core fucosylation of glycoproteins from human hepatocellular carcinoma and pancreatic ductal adenocarcinoma, resulting in the identification of 1130 and 782 core fucosylated glycosites, respectively. These results indicate that our STAGE method enables quantitative characterization of core fucosylation events from complex protein mixtures, which may benefit our understanding of core fucosylation functions in various diseases.

## Abstract (web summary)

Core fucosylation of N-linked glycoproteins has been linked to physiological and pathological processes. Here, the authors develop a mass spectrometry-based method that utilizes Endo F3 followed by PNGase F treatment to quantify site-specific glycoprotein core fucosylation in protein mixtures.

## Introduction

Protein glycosylation is one of the most abundant protein modifications in mammalian cells, which has been shown to be involved in various cellular activities, such as cell adhesion, cell-cell interactions, and protein folding^1–4^. Recently, core fucosylation has attracted great attention, found as one of the most critical modifications of N-linked glycans^5–7^. Studies have found that core fucosylation is associated with many physiological and pathological processes^5–12^. Certain core fucosylation of glycoproteins serves as more reliable cancer biomarkers than total protein expression level^7,8,13–20^. For instance, α-fetoprotein (AFP) is an FDA-approved biomarker for hepatocellular carcinoma (HCC), but it is also associated with other benign liver diseases^7,21^. On the other hand, an elevated level of core-fucosylated (CF) AFP is a signature for HCC, making it as a more specific biomarker for HCC^8,18^. Other reported CF-related markers include CF form of prostate-specific antigen and CF form of haptoglobin for prostate, aggressive prostate cancer, or pancreatic cancer, respectively^15,19,20,22,23^. Moreover, core fucosylation is also related to antibody-dependent cellular cytotoxicity, and removal of immunoglobulin G1 in its Fc region can significantly increase antibody-dependent cellular cytotoxicity^24–26^. Therefore, establishing a robust method for characterizing site-specific core fucosylation of glycoprotein would be beneficial for understanding the functions of core fucosylation and identifying the CF forms of glycoproteins for biomarker or therapeutic drug development.

Characterization of site-specific core fucosylation from complex protein mixtures is challenging. Recent advances in mass spectrometry (MS) technologies have enabled the analysis of intact glycopeptides^8^. Nonetheless, the unique CF glycosites identified in this type of study are still limited due to the low ionization efficiency of glycosylated peptides and heterogeneity of N-linked glycans at each glycosite. To identify CF glycopeptides from complex protein samples, intensive efforts have been focused on the enrichment of CF N-linked glycopeptides^8,27–30^. In particular, lectin affinity chromatography that binds specifically to CF glycans is widely used in the field for purifying CF glycopeptides^8,27–30^. Although lectin-based enrichment increases the identification rate of CF peptides, an additional enrichment method is still required to improve the overall glycosite coverage^8,29^. Thus, it is essential to establish a method enabling the isolation of CF peptides from complex mixtures efficiently to enhance CF glycosite coverage.

In this study, we present a method that employs sequential treatment of glycopeptides with enzymes (STAGE) to identify core fucosylation on glycosites from complex protein mixtures. Our method employs a mixed-mode polymeric sorbent, the Oasis MAX, to first enrich intact glycopeptides containing glycans with core fucosylation, non-core fucosylation, or both. The glycopeptide mixtures are then subjected to sequential enzymatic treatment with endo-beta-N-acetylglucosaminidase F3 (Endo F3) followed by PNGase F, resulting in partially or fully deglycosylated peptides with different mass signatures. Of note, Endo F3 cleaves CF biantennary glycans but shows no or minimal activity to CF tri-antennary, CF tetra-antennary oligosaccharides, and non-CF glycans (see Sigma-Aldrich product information for Endo F3, catalog number E2264). Thus, these glycans are released by subsequent PNGase F treatment to reduce the sample complexity, which in turn increases CF glycosite coverage. By utilizing basic reversed-phase liquid chromatography (bRPLC) to fractionate the partially and fully deglycosylated peptides, CF glycosite coverage is further improved. The established method is applied to study core fucosylation in HCC and pancreatic ductal adenocarcinoma (PDAC) tissues. These results demonstrate that our STAGE method can serve as a reliable quantitative tool for characterizing site-specific core fucosylation in glycoproteins from biological samples, and perhaps will increase our understanding of biological functions of core fucosylation in various diseases.

## Results

### Sequential treatment of glycopeptides with enzymes

**Fig. 1:**
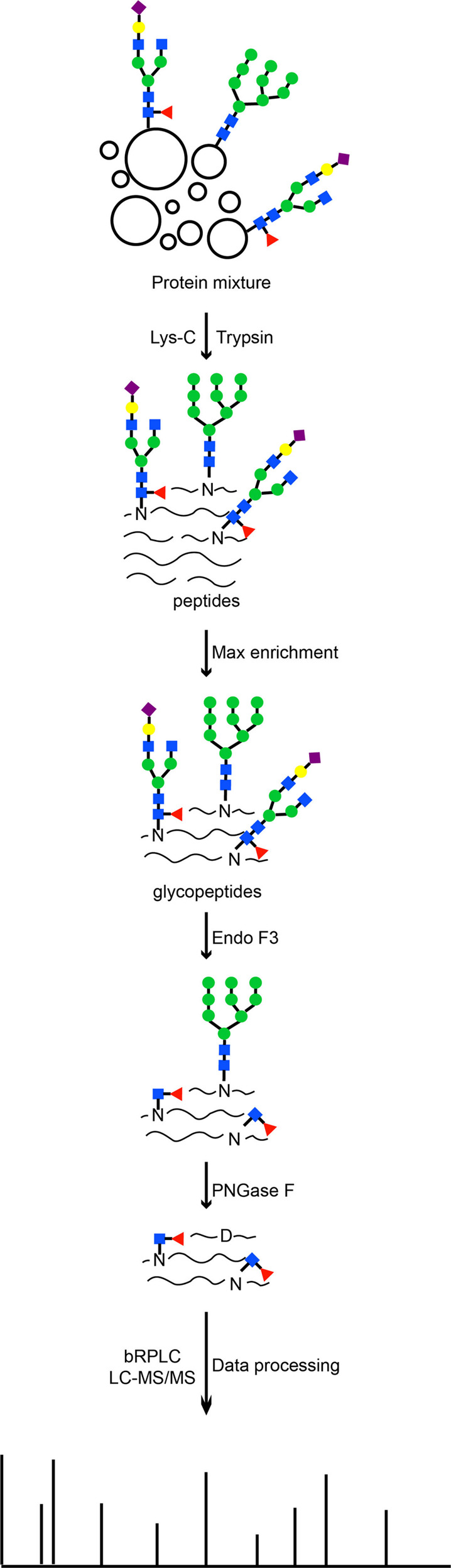
Schematic diagram of the workflow of the STAGE method. Blue square: *N*-acetylglucosamine, Green circle: mannose, Yellow circle: galactose, Red triangle: fucose, Purple diamond: sialic acid.

We have developed an MS-based method to quantitatively characterize core fucosylation from protein mixtures via STAGE. As shown in Fig. 1, in the STAGE method, sequential enzymatic treatments of Endo F3 followed by PNGase F were used to release different types of N-linked glycans from glycopeptides. This procedure resulted in partially and fully deglycosylated peptides with different mass signatures based on two types of N-linked glycosylation, namely core fucosylation and non-core fucosylation. Briefly, proteins and glycoproteins from biological or clinical samples were first digested into peptides by proteases, including Lys-C followed by trypsin. Next, intact glycopeptides were enriched from peptide mixtures. Glycopeptides were digested by endoglycosidase, Endo F3, to release CF biantennary glycan structures from glycopeptides, leaving only the core-fucosylated N-acetylglucosamine (N + 349 Da, referred to as Pep+HexNAc-Fuc) attached to the N-linked glycosite-containing peptides. Subsequently, the remaining N-linked glycans from intact glycopeptides were released by PNGase F to reduce the sample complexity derived from the heterogeneity of N-linked glycans, in which asparagine of the N-linked glycosites was converted to aspartic acid (*N* + 0.984 Da). The glycopeptides were fractionated with bRPLC and analyzed by LC-MS/MS, and then MS data were processed by proteomics software. Our developed strategy can reduce the complexity of peptide mixtures and quantitatively measure CF type of glycosylation at each glycosite from protein mixtures.

### Characterization of CF sites from liver tissues using STAGE

**Fig. 2:**
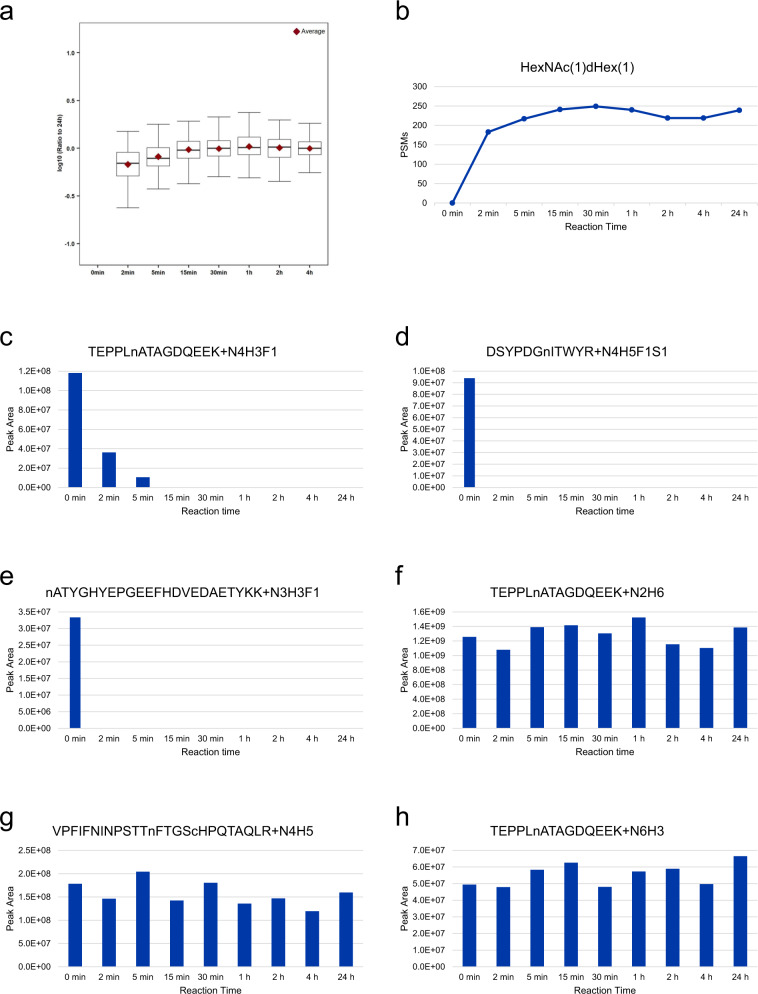
Assessment of the completeness of the enzymatic reaction using Endo F3. **a** Abundance ratio of each time point to 24 h for the core-fucosylated peptides (total *n* = 186 CF peptides; 0 min, NA; 2 min, *n* = 161; 5 min, *n* = 165; 15 min, *n* = 171, 30 min, *n* = 170; 1 h, *n* = 173; 2 h, *n* = 166; 4 h, *n* = 165). For each boxplot, the outline of the box denotes IQR with 25^th^ and 75^th^ percentiles, the solid line in the box indicates the median abundance ratio (center), and the whiskers outside of the box extend to the minimum and maximum abundance ratio. **b** The number of deglycosylated peptides containing residual HexNAc(1)dHex(1) that were identified at each time point. **c** The peak area of the CF biantennary glycopeptide (TEPPLnATAGDQEEK-N4H3F1, Uniprot ID: G3I973) from hypoxia upregulated protein 1 at different time points. **d** The peak area of the CF biantennary glycopeptide (DSYPDGnITWYR-N4H5F1S1, Uniprot ID: G3HRF8) from activated leukocyte cell adhesion molecule at different time points. **e** The peak area of the CF biantennary glycopeptide (nATYGHYEPGEEFHDVEDAETYKK-N3H3F1, Uniprot ID: G3ILN5) from reticulocalbin-3 at different time points. **f** The peak area of the non-CF glycopeptide (TEPPLnATAGDQEEK-N2H6, Uniprot ID: G3I973) from hypoxia upregulated protein 1 at different time points. **g** The peak area of the non-CF glycopeptide (VPFIFNINPSTTnFTGScHPQTAQLR-N4H5; Uniprot ID: G3HYW4) from lysosome-associated membrane glycoprotein 2 at different time points. **h** The peak area of the non-CF glycopeptide (TEPPLnATAGDQEEK-N6H3; Uniprot ID: G3I973) from hypoxia upregulated protein 1 at different time points. Core fucosylation was evidenced by the ion consisting of the peptide and the glycan moiety (HexNAc(1)dHex(1)). *c* carbomidomethylation of cysteine, *n* glycosylated site, *N* HexNAc, *H* Hex, *F* fucose, *S* sialic acid. Source data are provided as a Source Data file.

The STAGE method was first developed and benchmarked using CHO cells. We assessed the completeness of the enzymatic reaction by treatment of glycopeptides with Endo F3 under different reaction times (Fig. 2 and Supplementary Figure 1). The data showed that the Endo F3 reaction was completed within 30 min (Fig. 2a–e and Supplementary Figure 1). CF biantennary glycans are effectively released by Endo F3, while the enzyme has no or minimal activity on other glycan structures (Fig. 2f–h), in line with the manufacturer’s instructions (https://www.sigmaaldrich.com/deepweb/assets/sigmaaldrich/product/documents/155/114/e9762bul.pdf). In this study, we utilized 2 h as the reaction time for Endo F3 deglycosylation to ensure the completeness of the enzymatic reaction and control the false negative identification of CF glycopeptides. In addition, we evaluated the reproducibility of the entire STAGE workflow, including glycopeptide enrichment, Endo F3 deglycosylation, PNGase F deglycosylation, and quantification (Supplementary Data 1), and determined the average coefficient of variation (CV). A CV of 17.04%, 2.51%, 12.26%, and 5.54% was achieved for glycopeptide enrichment, Endo F3 deglycosylation, PNGase F deglycosylation, and quantification from TMT labeling, respectively, suggesting good reproducibility of the STAGE workflow. Good reproducibility of the STAGE workflow for quantification of CF glycosites was achieved when the workflow was applied to characterize site-specific core fucosylation of bovine serum fetuin as well as the protein mixture of CHO cells (Supplementary Data 1). We also applied the method to characterize site-specific core fucosylation of well-known standard glycoproteins, including bovine serum fetuin and RNase B from bovine pancreas, revealing core fucosylation in two glycosites in fetuin and no core fucosylation in RNase B (Supplementary Data 2), in line with previous studies^31,32^. The method requires only 20 μg of starting material for the identification of the CF glycosites in standard glycoprotein fetuin (Supplementary Data 3).

We then applied the method to HCC tumors and normal tissues. All glycosites identified by SEQUEST^33^ were filtered by the presence of consensus motif (N-X-S/T, X can be any amino acid residue except proline) prior to the downstream analyses. To further investigate whether the resulting sites were CF glycopeptides containing Pep+HexNAc-Fuc modification, the spectra assigned to these glycopeptides were assessed for the existence of at least one of the four oxonium ions at m/z 126.055, m/z 138.055, m/z 186.066, and m/z 204.087^28,34^. Oxonium ions at m/z 126.055, m/z 138.055, m/z 186.066, and m/z 204.087 were found in 1127, 1112, 1069, and 1118 of CF sites, respectively, corresponding to 1102 CF peptides. Supplementary Figure 2 shows MS/MS spectra of a peptide, LHNQLLP*N*^511^VTTVER from glutathione hydrolase 1 proenzyme (GGT1, UniProt: P19440) modified by CF biantennary glycans (Pep+HexNAc-Fuc, Endo F3-modified). When the N-glycans on the glycosite (*N*^511^) were cleaved by Endo F3, the oxonium ions were detected in the lower mass range, indicating attachment of monosaccharide residue(s) to the peptide (Supplementary Figure 2). The b- and y-ions as well as the ions of neutral loss provided additional information on the position of CF (loss of HexNAc-Fuc, Supplementary Figure 2).

**Fig. 3:**
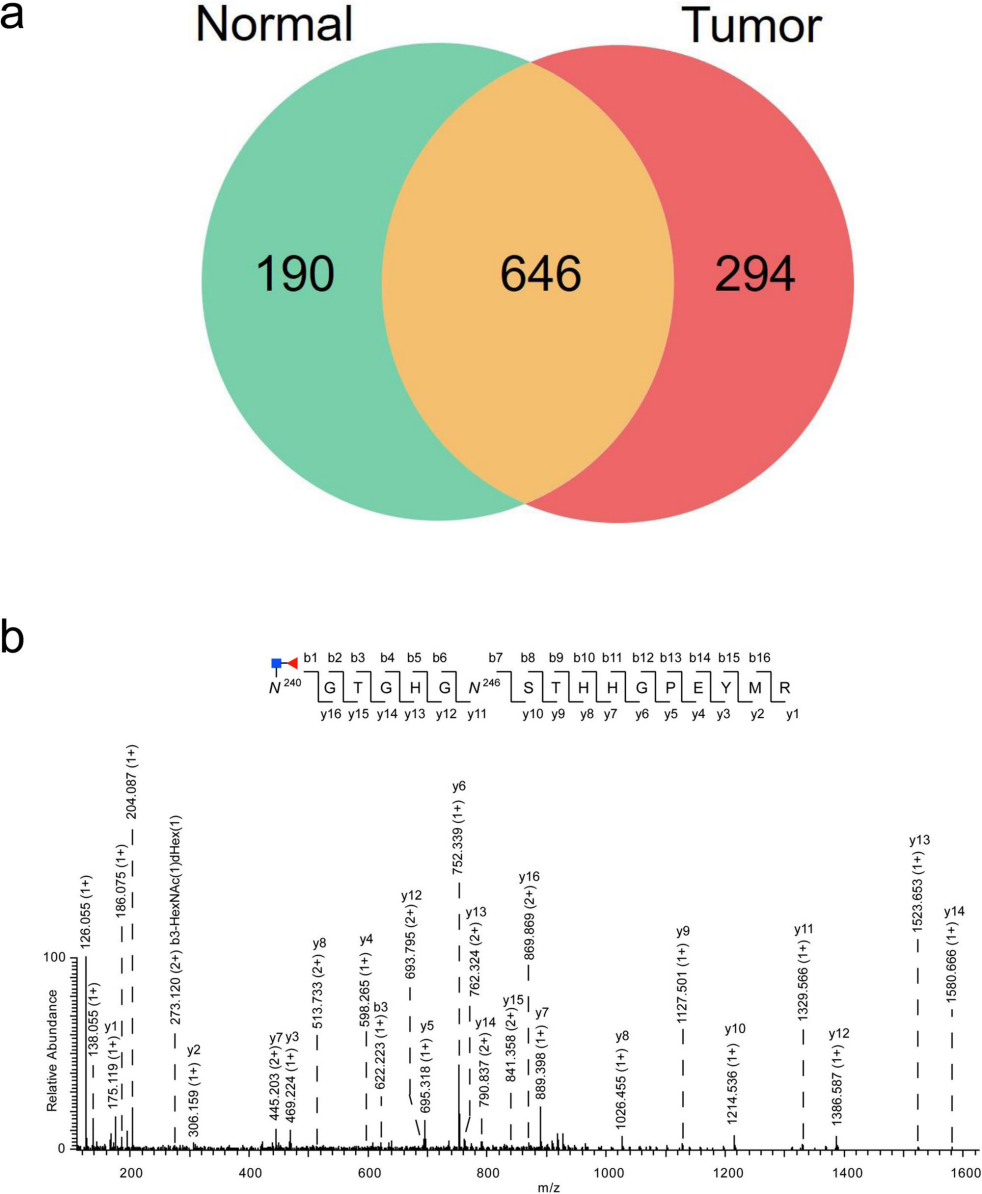
Characterization of CF sites from human HCC tumor and normal tissues using the STAGE method. **a** Number of identified CF sites in tumor and normal liver tissues. **b** MS/MS spectrum of the CF glycopeptide, *N*^240^GTGHG*N*^246^STHHGPEYMR from hemopexin (UniProt ID: P02790). The site, *N*^240^, was modified by CF biantennary glycans, whereas the site, *N*^246^, was a glycosite that was modified by other glycans. Keys: b-HexNAc(1)dHex(1) represents a disaccharide, HexNAc(1)dHex(1), is lost from this b ion; y-HexNAc(1)dHex(1) represents a disaccharide, HexNAc(1)dHex(1), is lost from this y ion; b-HexNAc(1) represents a monosaccharide residue, HexNAc(1), is lost from this b ion; y-HexNAc(1) represents a monosaccharide residue, HexNAc(1), is lost from this y ion.

Using the STAGE method, a total of 1130 unique CF glycosites (Pep+HexNAc-Fuc) were identified from human liver tumors and normal tissues (Fig. 3a and Supplementary Data 4). Furthermore, our proposed method was also capable of capturing glycopeptides containing multi-glycosylation sites that simultaneously occurred on the same glycopeptides. We identified 68 non-redundant CF-containing glycopeptides with multiple glycosylation sites and 49 and 57 CF-containing glycopeptides identified from normal and tumor tissues, respectively (Supplementary Data 5). Figure 3b shows a spectrum of one of the glycopeptides with multiple glycosylation sites as an example. The glycopeptide (*N*^240^GTGHG*N*^246^STHHGPEYMR) from hemopexin (UniProt ID: P02790) contained two glycosites in which *N*^240^ was modified by CF biantennary glycans and *N*^246^ was modified by other glycans. The two glycosites were distinguished from each other by examining the b- and y-ions, whereas the oxonium ions at m/z 126.055, m/z 138.055, m/z 186.076, and m/z 204.087 were used to validate the attachment of monosaccharide residues to the peptide.

### Analysis of consensus motif for CF glycosites

**Fig. 4:**
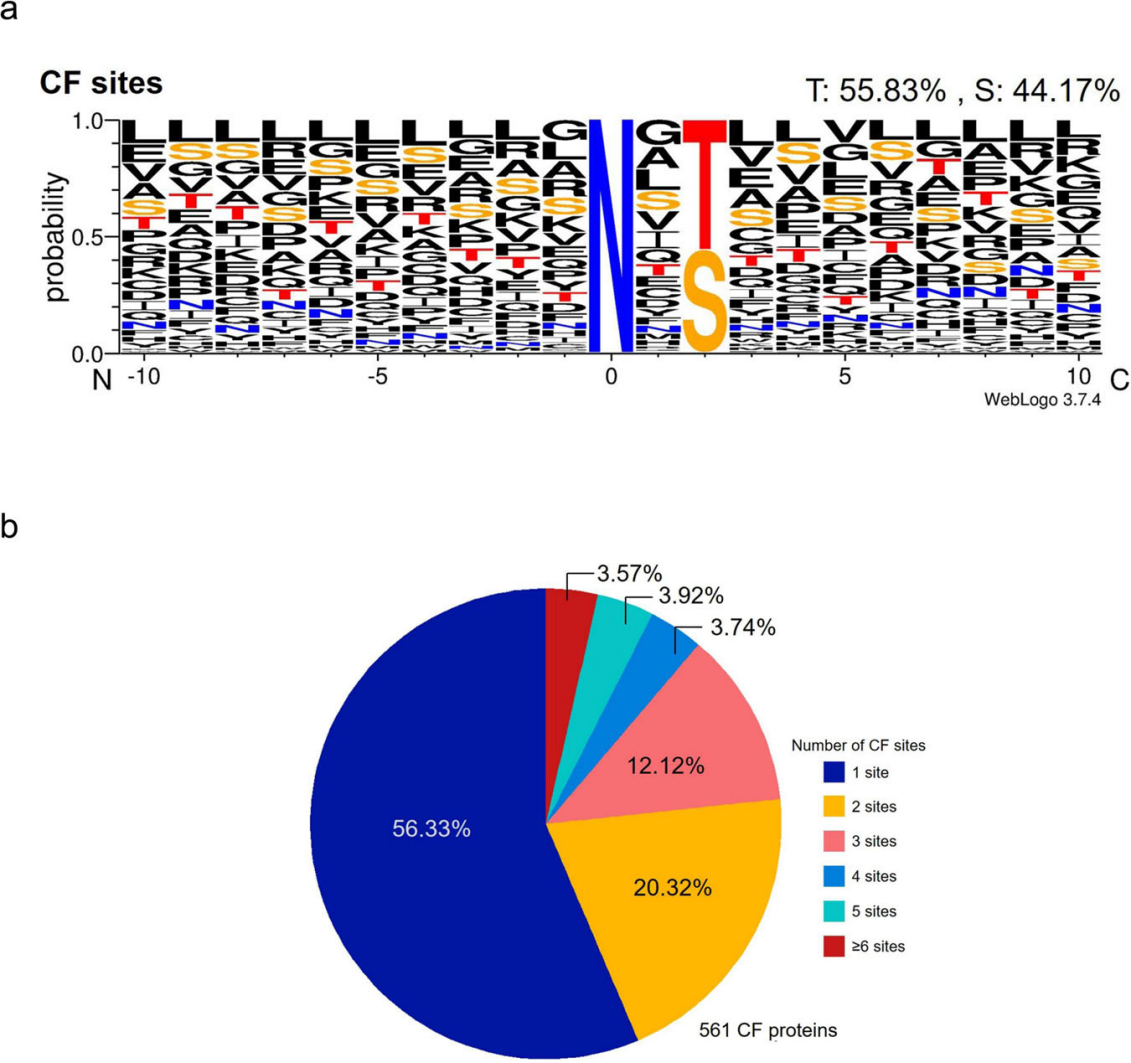
Analysis of consensus motifs for CF glycosites identified and distribution of CF glycosites on CF site-contained glycoproteins by the STAGE method. **a** Relative frequency of ±10 amino-acid residues around the asparagine of N-linked glycosylation site (fixed at the center position of 0) was obtained by using flanking sequences derived from all CF sites consisting of Pep+HexNAc-Fuc identified using the STAGE method. Threonine (T, red) is the most frequently observed amino acid residue in position 2 for CF sites. *N* = Asparagine (blue), S = Serine (yellow), and the rest of the amino acid residues are in black. **b** Distribution of CF site-contained glycoproteins (CF proteins^#^) with ≥1 CF sites that were identified by the STAGE method. ^#^CF proteins: glycoproteins had at least one CF site identified.

It is well-known that N-linked glycosylation has a consensus motif composed of N-X-S/T, where X at the +1 position is any amino acid residue except proline. Typically, threonine (T) at +2 position is more frequently observed on the motif relative to serine (S)^35^. To explore if CF glycosites behaved differently from general glycosites, we generated flanking sequences composed of 21 amino-acid residues that asparagine on the glycosylation motif was fixed at the center (position 0). As shown in Fig. 4a, we observed that T is more frequently found in position 2 compared to S with a frequency rate of 55.83% for CF sites (Fig. 4a), in line with the results of overall glycosylation sites present in the previous study^35^.

The 1130 CF sites identified from tumor and normal tissues originated from 1102 CF glycopeptides corresponding to 561 CF site-contained proteins (CF proteins, Supplementary Data 6). Among the 561 CF proteins, 56.33% had only one CF site (Fig. 4b). There were 20.32%, 12.12%, and 3.74% CF proteins containing 2, 3, and 4 CF glycosites, respectively. Approximately 7.49% of CF proteins had more than four CF sites. To estimate the performance of our method for studying core fucosylation events on large-scale glycoproteomics, we compared our results to those previously reported in the literature^9,28,34,36^. Although different specimens were used, on average approximately 500 CF sites were identified in the previous studies^9,28,34,36^, while a total of 1130 CF sites were identified using the STAGE method, indicating STAGE is a robust approach to characterize CF sites.

### Quantitative analysis of CF glycoproteins in HCC tissues

An elevated level of core fucosylation of AFP is an FDA-approved biomarker for HCC^18^. Using the STAGE method, we expanded the characterization of core fucosylation from HCC tissues using a TMT-based quantitation approach^37,38^. After labeling of peptides, the TMT-labeled peptides from different samples were combined. Intact glycopeptides were then enriched, deglycosylated with Endo F3 followed by PNGase F, and fractionated into 12 fractions. Each was subjected to LC-MS/MS analysis; a total of 366 CF glycosites from 369 CF glycopeptides (with ≤2 missed cleavages) were quantified (Supplementary Data 7). The reference channel (TMT-126) was generated by pooling equal amounts of proteins from HCC tumor and normal tissues and used for normalization across the TMT set. TMT reporter channels from TMT-127N to TMT-128C were assigned to normal tissues (Supplementary Data 7). HCC tumor tissues were labeled from TMT-129N to TMT-130N (Supplementary Data 7).

**Fig. 5:**
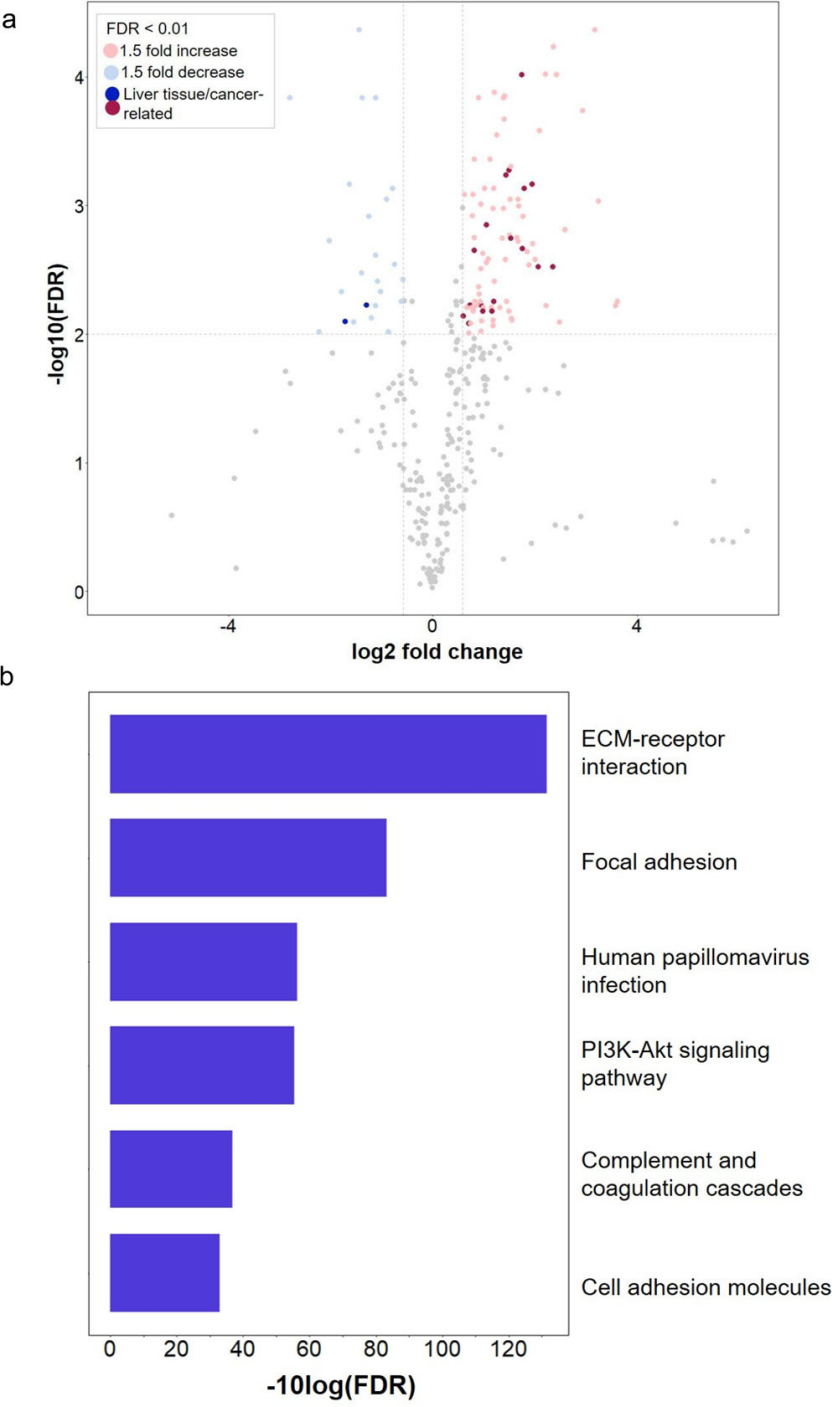
Quantitative analysis of glycosites from HCC tumor (Tumor) and normal (Normal) tissues by TMT labeling coupled with the STAGE method. **a** Differential expression of glycosylation sites containing ≥1 CF sites between Tumor and Normal tissues. Glycosylation sites that were up- and down-regulated (FDR < 0.01 and fold change ≥1.5) in tumor relative to normal tissues are highlighted in pink and blue, respectively, of which glycosites from liver tissue/cancer-related glycoproteins are highlighted in darker colors. **b** KEGG pathway assignment based on upregulated proteins. Source data are provided as a Source Data file.

As we examined the glycopeptides containing CF sites quantified in >75% of TMT reporter channels (Supplementary Data 8), we observed 88 and 24 CF glycosylation events that were up- and down-regulated in tumor tissues (FDR < 0.01 and fold change ≥1.5) relative to normal, respectively (Fig. 5a and Supplementary Data 9). Seventy-two CF glycosylation sites were also detected by HCC label-free data (Supplementary Data 4), and the differential expression of 52 CF glycosites were further validated in the label-free data (Supplementary Data 10), suggesting the reliability of our reported tumor-associated CF glycosylation events. In particular, the glycosite (*N*^603^) of EGFR was upregulated in HCC tumors relative to normal in TMT-labeled (Supplementary Data 9). This glycosite of EGFR was in one of the domains that were reported to be essential for the activation of EGFR^39^. Among the differentially expressed glycosites, 5 CF sites were originated from four HCC-related proteins (Fig. 5a and Supplementary Data 9), such as epidermal growth factor receptor (EGFR), myeloperoxidase (MPO), and slit homolog 2 protein (SLIT2). By performing KEGG pathway enrichment analysis using *WebGestalt*^40^, we found pathways related to cell adhesion and human papillomavirus (HPV) infection as well as PI3K-Akt signaling pathway were enriched by upregulated CF glycosylation events (Fig. 5b, Supplementary Data 11). Notably, activation of PI3K-Akt signaling pathway was found to be involved in cancer progression via promoting proliferation and increasing cell survival^41^, whereas HPV infection has been shown to contribute to other cancer types, including cervical cancer^42^ and head and neck cancers^43^, there have been no reports linking HPV to liver cancer. Instead, hepatitis B/C infections have been associated with liver cancer^44,45^, and these observed features may be representative of viral infection contributing to oncogenesis in a variety of cancer subtypes.

### Characterization of site-specific core fucosylation in PDAC

**Fig. 6:**
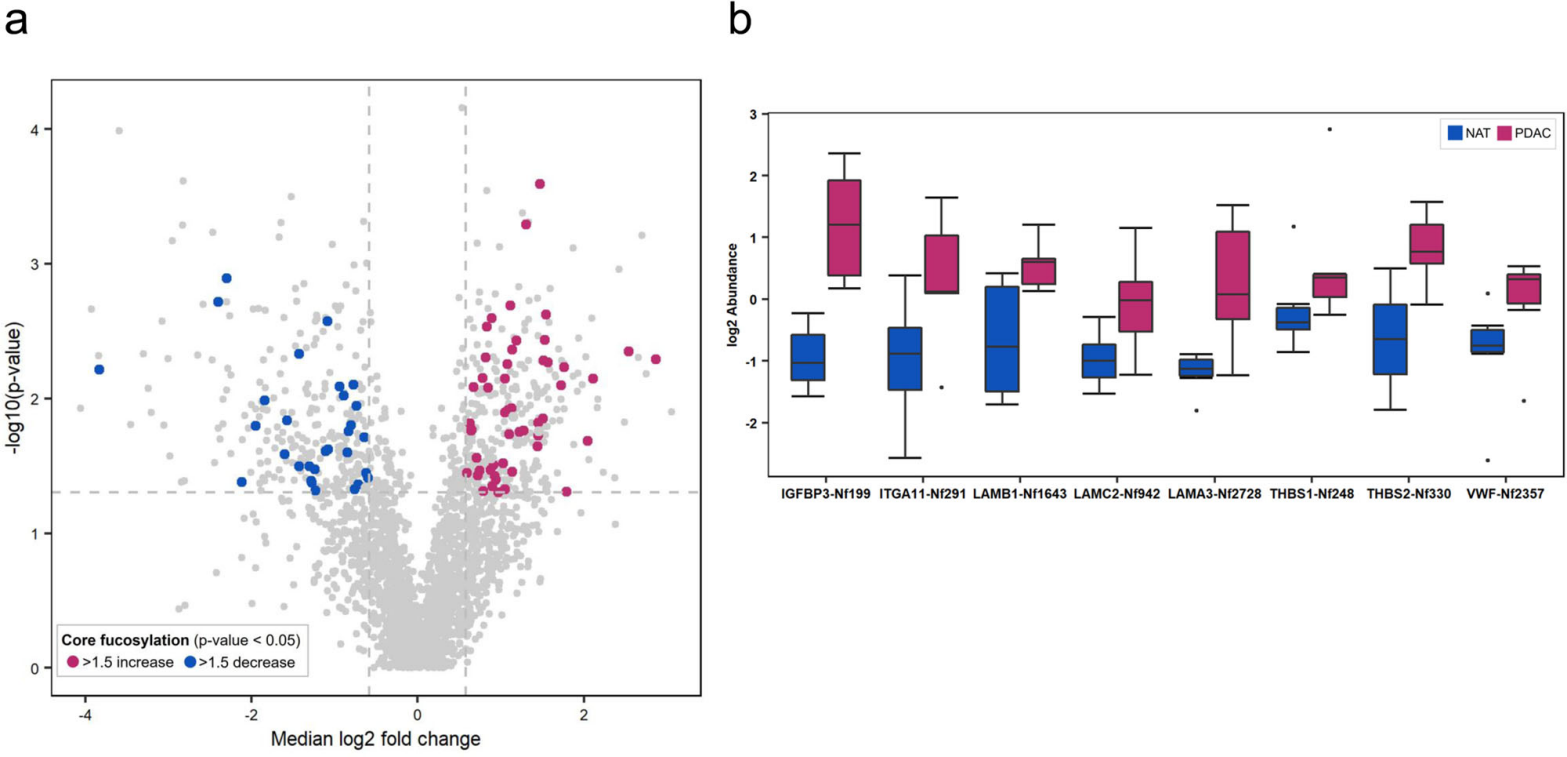
Differential analysis between six PDAC tissues and the paired NATs. **a** A total of 551 differentially expressed glycosites were identified, including 54 upregulated (red dots) and 31 downregulated CF glycosites (blue dots) in PDACs relative to paired NATs (paired *t* test, *p* < 0.05, and fold change >1.5). **b** The expression profiles of the CF glycosites that are differentially expressed between PDACs (*n* = 6) and NATs (*n* = 6). For each boxplot, the outline of the box denotes IQR with 25th and 75th percentiles, the solid line in the box indicates median abundance ratio (center), and the whiskers outside of the box extend to the minimum and maximum abundance ratio. Source data are provided as a Source Data file.

PDAC is one of the deadliest of all solid malignancies with a five-year survival rate >10%^46^, and is associated with core fucosylation of glycoproteins^47^. To this end, we characterized site-specific core fucosylation of 6 paired tumor/normal samples from patients with PDAC, resulting in the identification of 782 CF glycosites from 860 glycopeptides (with ≤2 missed cleavages) across 6 paired samples (Supplementary Data 12). By examining glycopeptides quantified in >75% of the paired samples (Supplementary Data 13), we found 54 upregulated and 31 downregulated CF glycosites in PDACs relative to paired normal adjacent tissues (NATs) (paired *t* test, *p* < 0.05, and fold change >1.5, Fig. 6a, Supplementary Data 14). In particular, up-regulation of core fucosylation on the proteins involved in the PI3K-Akt signaling pathway, e.g., LAMB1-N1643, LAMC2-N942, LAMA3-N2728, THBS1-N248, and THBS2-N330, was observed, suggesting potential functional roles of core fucosylation in the regulation of this oncogenic signaling pathway in PDAC^48^ (Fig. 6b). In addition, PDAC-associated proteins (IGFBP3 and ITGA11) displayed upregulated core fucosylation at the glycosylation sites N199 and N291, respectively^49,50^ (Fig. 6b). As a secreted glycoprotein, upregulation of core fucosylation at the site N199 of IGFBP3 could serve as a potential marker for early detection of PDAC^51^. Interestingly, core fucosylation of von Willebrand factor (VWF) at the glycosylation site N2357 was significantly upregulated (>1.5-fold, *p* < 0.05, Fig. 6b) in PDACs relative to NATs, while total protein expressions of VWF were comparable between tumors and NATs according to the previous study^51^. Although VWF can promote pro-inflammatory signaling, and regulate angiogenesis and vascular permeability, which may facilitate tumor cell growth and extravasation across the vessel wall^52^, little is known about the role of core fucosylation at this glycosylation site and its effect on VWF function. Of note, we observed different levels of CF among individuals in both PDAC tumors and adjacent normal tissues (Supplementary Data 13). Further investigations with larger sample size are warranted to verify these CF changes.

## Discussion

We have developed the STAGE method that utilizes sequential enzymatic treatments to introduce different mass signatures for characterizing glycosites occupied by CF N-linked glycans. The STAGE method also employs glycopeptide enrichment via Oasis Max to reduce sample complexity before enzymatic treatments. The resulting CF peptides (Pep+HexNAc-Fuc) are subjected to LC-MS/MS analysis, followed by data processing using a standard proteomics pipeline. By incorporating quantitative proteomics, we were able to further quantitatively examine the impact of core fucosylation under different biological conditions (Figs. 5, 6).

Given the complexity of N-linked glycosylation, we believe that the STAGE method will enhance the quantitative analysis of a large number of CF glycosites for investigating functional roles of core fucosylation in regulating signaling pathways related to cancer biology^5–7,53^. Indeed, we detected 1130 unique core fucosylation sites from label-free HCC tumors and normal samples, which showed improved analytical coverage of core-fucosylated glycosites compared to the previous studies^9,28,34,36^ (Fig. 3a). Among the 1130 CF glycosites, 96 CF glycosites were identified along with ≥1 PNGase F-modified glycosites in the same glycopeptides (Supplementary Data 5), which were rarely detected in the previous studies^27,36,54^.

The incorporation of TMT labeling in the STAGE method enabled us to identify differential expression of core fucosylation events between HCC tumors and normal samples. In particular, the glycosite (*N*^603^) of EGFR was upregulated in HCC tumors relative to normal (Fig. 5a and Supplementary Data 9), which was in one of the domains that were reported to be essential for the activation of EGFR^39^. The EGFR signaling pathway has been identified as a key player in HCC development^55^. In addition, upregulated CF glycosylation events in HCC tumor was associated with PI3K-Akt signaling pathway (Fig. 5b and Supplementary Data 11), which is responsible for initiating and promoting HCC^56^. Thus, selective inhibition of the glycosylation enzymes that are responsible for core fucosylation, e.g., α1,6-fucosyltransferase (FUT8), will likely attenuate increased core fucosylation that was found on these glycoproteins in HCC tumor and serves as a potential therapeutic strategy for HCC.

Although, in the present study, our proposed method was applied to the investigation of the differences in core fucosylation between tumor and normal tissues from HCC and PDAC, the method is suitable for analyzing and characterizing core fucosylation in other biological or clinical samples under different conditions, such as cell, serum, urine, and other body fluid samples. It can also be used to characterize core fucosylation of biotherapeutics, for example, IgG1, where core fucose has an impact on the therapeutic functions^7,57^.

One limitation of the strategy is the specificity of the Endo F3 enzyme. Endo F3 is not able to release CF N-linked glycans with tri- and tetra-antennary present on glycosites according to the manufacturer’s instructions (https://www.sigmaaldrich.com/deepweb/assets/sigmaaldrich/product/documents/155/114/e9762bul.pdf). These types of N-glycans are released by subsequent PNGase F treatment in the STAGE method. Therefore, CF glycans from biantennary glycosites can be released by Endo F3, identified, and quantified by their specific mass tags (Pep+HexNAc-Fuc) in tumor and normal tissues of HCC and PDAC to characterize core fucosylation changes related to these cancer types.

## Methods

### Human Subjects

HCC samples were obtained from The Johns Hopkins Hospital (JHH). Four HCC tumors and 4 normal liver tissues, as well as 6 PDAC tumors and six, paired PDAC NATs were carefully evaluated histologically and included in this study. The donors have given informed consent for their samples being used for research. All human specimens were existing specimens and subjects cannot be identified. The study was exempted from human subject research based on Category 4 - Secondary Research (§46.101(b)(4)). Participants did not receive financial compensation.

### Tissue samples

Four HCC tumors and four normal liver tissues were included, composed of two pairs of tumor and tumor-matched NATs, two NATs without paired tumor tissues, and two tumor tissues without paired normal tissues (HCC tumor sample donors: 2 men, 2 women/age-range: 36–70/tumor size: 3.8–15.2 cm/tumor grade: G1-G2 / pathological stage: pT1-pT3; NAT sample donors: 2 men, 2 women/age-range: 36–69/tumor size, grade and pathological stage: not applicable). The specimens were collected from surgically resected liver samples. All specimens were snap-frozen and stored at −80 °C until use. The hematoxylin and eosin (H&E) stained sections were reviewed by an American Pathology Board-certified pathologist (Q.K.L.) to ensure the representation of the tumor and normal area.

Similarly, in the study of PDAC, 6 PDAC tumors and paired NATs were carefully evaluated histologically and processed according to Clinical Proteomic Tumor Analysis Consortium (CPTAC) guidelines (donors: 4 men, 2 women/age-range: 42–75/BMI range: 16–30.12/tumor site and focality: 5 heads and 1 tail, all unifocal/tumor size range: 2.8–5 cm/pathological stage: IB-IV).

### The Chinese hamster ovary (CHO) cell culture

The CHO cell line was a gift from Dr. Michael J Betenbaugh (Johns Hopkins University)^58–60^. The CHO cell line was cultured in McCoy’s 5 A Medium (Gibco) with 10% FBS, and 100 U/mL of Penicillin-Streptomycin (Gibco).

### Protein preparation

Chilled lysis buffer (8 M urea, 75 mM NaCl, 50 mM Tris (pH 8.0), 1 mM EDTA, 2 µg/ml aprotinin, 10 µg/ml leupeptin, 1 mM PMSF, 1:100 (vol/vol) Phosphatase Inhibitor Cocktail 2, 1:100 (vol/vol) Phosphatase Inhibitor Cocktail 3, 10 mM NaF, and 20 µM PUGNAc) was added to tissues/cell pellets. The resulting mixture was vigorously vortexed for 20 seconds, followed by incubation on ice for 15 minutes. This cycle was repeated three times. Cell debris was removed by centrifugation at 18,000 × *g* for 10 minutes at 4 °C. Protein concentration was measured with a BCA assay using supernatant.

### Enzymatic digestion

One milligram of proteins from tissues, cells, or standard glycoproteins, including bovine serum fetuin (Millipore Sigma) and bovine pancreas RNase B (Millipore Sigma), were reduced and alkylated with 5 mM dithiothreitol (37 °C, 1 hour) and 10 mM iodoacetamide (25 °C, 45 minutes, in the dark), respectively. The denatured proteins were first digested by Lys-C (FUJIFILM Wako Chemicals USA. Corporation) in an enzyme to substrate ratio of 1:50 for 2 hours at 25 °C, followed by tryptic digestion (Promega) in an enzyme to substrate ratio of 1:50 for 14 hours at 25 °C. The enzymatic reaction was terminated by adding 50% of formic acid (final pH <3). The reaction solution was then centrifuged at 16,000 × *g* for 10 minutes at 4 °C.

### C18 desalting

The digested peptides derived from 1 mg of proteins were desalted with C18 solid-phase extraction (100 mg Sorbent per Cartridge, Waters tC18 SepPak). The cartridge was conditioned by acetonitrile (ACN), 50% ACN (0.1% FA), and 0.1% TFA. The peptides were loaded twice. The cartridge was washed with 0.1% TFA and 1% FA to remove salts. The peptides were eluted with 50% ACN (0.1% FA).

### Tandem Mass Tag (TMT) labeling

TMT labeling of peptides was conducted according to manufacturer instructions. In brief, 100 μg of peptides were resuspended in 50 mM HEPES buffer (pH 8.5) at the final concentration of 5 µg/µl. TMT reagent (Thermo Fisher Scientific, 10 plex, 1 × 0.8 mg) was dissolved in anhydrous acetonitrile (Sigma-Aldrich) and added to the peptide solution. The mixture was incubated at room temperature with a vortex (1000 rpm) for 1 hour. The reaction was quenched by adding 5% hydroxylamine to the solution. The TMT-labeled peptides were mixed together for glycopeptide enrichment.

### Glycopeptide enrichment

The glycopeptides were enriched with MAX solid-phase extraction^61,62^ (Waters) from tryptic peptides or TMT-labeled tryptic peptides from above. The MAX cartridge was conditioned with ACN, 100 mM triethylammonium acetate buffer, water, and 95% ACN (0.1% TFA). The peptides were loaded twice. The cartridge was washed with 95% ACN (0.1% TFA) to remove non-glycosylated peptides. The glycopeptide fraction was eluted out by using 50% ACN (0.1% TFA).

### Assessment of the completeness of enzymatic reaction

Approximately 80 μg of glycopeptides of CHO cells were treated with 5 μl of Endo F3 (~5 milliunits/μl, Millipore Sigma) for 2 min, 5 min, 15 min, 30 min, 1 h, 2 h, 4 h, and 24 h. For the experiment of 0 min, no enzyme was added to the peptides. The resulting peptides were subjected to LC-MS/MS.

### Deglycosylation

N-linked glycans were digested by Endo F3 (Millipore Sigma) followed by PNGase F (New England BioLabs). First, label-free or TMT-labeled glycopeptides were resuspended in 100 mM ammonium acetate (pH 5.5) and treated with Endo F3 at an enzyme/peptides ratio of 25 milliunits/80 μg of glycopeptides (50 milliunits/80 μg of glycopeptides for HCC tumor and normal tissues). The enzymatic reaction was conducted at 37 °C for 2 hours. The samples were dried in a speed vacuum centrifuge for 5 hours. The dried peptides were redissolved in 100 mM ammonium bicarbonate (pH 8) and treated with PNGase F (New England BioLabs) at an enzyme/peptides ratio of 3000 New England Biolabs units/80 μg of glycopeptides. The reaction solution was incubated at 37 °C for 2 hours. The resulting peptides were desalted with C18 solid-phase extraction.

### Evaluation of reproducibility

A large batch of the proteins from CHO cells was digested with Lys-C followed by trypsin and divided into three aliquots. Glycopeptides from each aliquot were enriched by MAX solid-phase extraction (Waters), and then 5 μg of glycopeptides from each aliquot were taken out for LC-MS/MS analysis to evaluate the reproducibility of glycopeptide enrichment. The remaining glycopeptides from three aliquots were combined and then divided into three aliquots. Each aliquot was treated with Endo F3, and then 5 μg of deglycosylated peptides were taken out from each aliquot for LC-MS/MS analysis to assess the reproducibility of Endo F3 treatment. The remaining deglycosylated peptides were combined and then divided into three aliquots. Each aliquot was treated with PNGase F, and then 5 μg of deglycosylated peptides were taken out from each aliquot for LC-MS/MS analysis to assess the reproducibility of PNGase F treatment. The remaining deglycosylated peptides were combined, divided into three aliquots, labeled by TMT reagent, and subjected to LC-MS/MS analysis for evaluation of reproducibility of quantification.

To evaluate the reproducibility of the entire STAGE workflow, fetuin and the protein mixture from CHO cells were digested with Lys-C followed by trypsin. The peptides were then divided into three aliquots. Glycopeptides from each aliquot were enriched by MAX solid-phase extraction, treated with Endo F3 followed by PNGase F, labeled by TMT reagent, and subjected to LC-MS/MS analysis.

### Limitation of detection assessment

A large batch of fetuin protein was digested with Lys-C followed by trypsin. The peptide concentration was measured with a BCA assay. Different amounts of peptides (0.2 μg, 1 μg, 4 μg, 20 μg, 100 μg, 200 μg, and 500 μg) were taken out and treated with Endo F3 followed by PNGase F. The resulting deglycosylated peptides were subjected to LC-MS/MS analysis for evaluation of limitation of detection.

### Peptide fractionation by bRPLC

The TMT-labeled and unlabeled deglycosylated peptides of HCC tumor and normal tissues were fractionated with a 4.6 mm × 100 mm Zorbax ExtendC18 analytical column (1.8 μm particles, Agilent Technologies) lined up with a 1220 Series HPLC (Agilent Technologies). Buffer A and B are 5 mM ammonium formate in 2% ACN (pH 10) and 5 mM ammonium formate in 90% ACN, respectively. Samples were separated by using a 120-minute gradient: 0-10 minutes 0% B, 10–15 minutes 0–8% B, 15–100 minutes 8-35% B, 100–105 minutes 35–95% B, and 105–120 minutes 95% B. The flow rate was set at 0.3 mL/minute. Ninety-six fractions were collected into a 96-well plate from 16 to 111 minutes. These fractions were pooled every 12 fractions (e.g., fractions 1, 13, 25, 37, 49, 61, 73, and 85 were combined). The resulting 12 fractions were dried in a speed vacuum centrifuge and stored at −80 °C until LC-MS/MS analysis.

The TMT-labeled peptides of PDAC were fractionated with a 4.6 mm × 250 mm Zorbax ExtendC18 analytical column (3.5 μm particles, Agilent Technologies) lined up with a 1220 Series HPLC (Agilent Technologies). Buffer A and B are 5 mM ammonium formate in 2% ACN (pH 10) and 5 mM ammonium formate in 90% ACN, respectively. Samples were separated by using a non-linear gradient: 0% buffer B (7 min), 0% to 16% buffer B (6 min), 16% to 40% buffer B (60 min), 40% to 44% buffer B (4 min), 44% to 60% buffer B (5 min) and then held at 60% buffer B for 14 min. Ninety-six fractions were collected into a 96-well plate. Collected fractions were concatenated into 24 fractions^51,63^. Eight percent of each of the 24 fractions was aliquoted for global proteomics characterization. The remaining 92% of the sample was further concatenated into 12 fractions for phosphopeptide enrichment followed by glycopeptide enrichment using MAX solid-phase extraction^61,64^.

Of note, each fraction of TMT-labeled or label-free peptides was subjected to single-shot LC-MS/MS analysis, except for label-free deglycosylated peptides of HCC tumor and normal samples, which were subjected to LC-MS/MS analyses in triplicate.

### Nano LC-MS/MS analysis

Unlabeled deglycosylated peptides of HCC tumor and normal tissues were analyzed on a Q Exactive HF-X Hybrid Quadrupole-Orbitrap Mass Spectrometer (Thermo Scientific). Approximately 1 ug of deglycosylated peptides were loaded onto an in-house packed C18 column (75 μm × 28 cm, (1.9 μm Reprosil-Pur C18-AQ beads (Dr. Maisch GmbH)) lined up with an EASY-nLC 1200 (Thermo Fisher Scientific). For unlabeled peptides, the flow rate was set at 300 nl/min. Buffer A and B were 3% ACN (0.1% FA) and 90% ACN (0.1% FA), respectively. A 130-minute gradient was deployed, consisting of the following steps: 0–1 minute 2% B, 1–109 minutes 2–30% B, 109–114 minutes 30–60% B, 114–115 minutes 60–90% B, 115–120 minutes 90% B, 120–121 minutes 90–50% B, and 121–130 minutes 50% B. Peptides were eluted from the column and nanosprayed directly into the mass spectrometer. The mass spectrometer was operated in a data-dependent mode. Parameters were set as followed: MS1 resolution 60,000, AGC target 1e6, maximum IT 60 ms, scan range 400 to 1800 m/z, dynamic exclusion 15 s, charge inclusion 2–6, top 20 ions selected for MS2; MS2 resolution 15,000, AGC target 5e5, and a normalized collision energy (NCE) of 33.

TMT-labeled deglycosylated peptides of HCC tumor and normal tissues were analyzed on a Q Exactive HF-X Hybrid Quadrupole-Orbitrap Mass Spectrometer (Thermo Scientific). The flow rate was set at 200 nl/minute. A 110-minute gradient was deployed, consisting of the following steps: 0–1 minute 2–6% B, 1–85 minutes 6–30% B, 85–94 minutes 30–60% B, 94–95 minutes 60–90% B, 95–100 minutes 90% B, 100–101 minutes 90–50% B, and 101–110 minutes 50% B. Peptides were eluted from the column and nanosprayed directly into the mass spectrometer. The mass spectrometer was operated in a data-dependent mode. Parameters were set as followed: MS1 resolution 120,000, AGC target 3e6, maximum IT 50 ms, scan range 350 to 1500 m/z, dynamic exclusion 20 s, charge inclusion 2–5, top 20 ions selected for MS2; MS2 resolution 45,000, AGC target 1e5, and an optimized normalized collision energy (NCE) of 33.

Unlabeled deglycosylated peptides of CHO cells, as well as standard glycoproteins (i.e., fetuin and RNase B), were analyzed on an Orbitrap Fusion Lumos mass spectrometer (Thermo Scientific). The peptides were separated on an in-house packed C18 column (75 μm × 28 cm, (1.9 μm Reprosil-Pur C18-AQ beads (Dr. Maisch GmbH)) lined up with an EASY-nLC 1200 (Thermo Fisher Scientific). The flow rate was set at 200 nl/min. Buffer A and B were 3% ACN (0.1% FA) and 90% ACN (0.1% FA), respectively. The peptides were separated with a 6–30% B gradient in 84 min. Peptides were eluted from the column and nanosprayed directly into the mass spectrometer. The mass spectrometer was operated in a data-dependent mode. Parameters were set as followed: MS1 resolution 60,000, AGC target 4e5, maximum IT 50 ms, scan range 350 to 2000 m/z, dynamic exclusion 45 s, charge inclusion 2–6, cycle time 2 s; MS2 resolution 15,000, AGC target 5e4, maximum IT 50 ms, and an NCE of 34.

Unlabeled intact glycopeptides of CHO cells were analyzed on an Orbitrap Fusion Lumos mass spectrometer (Thermo Scientific). The peptides were separated on an in-house packed C18 column (75 μm × 28 cm, (1.9 μm Reprosil-Pur C18-AQ beads (Dr. Maisch GmbH)) lined up with an EASY-nLC 1200 (Thermo Fisher Scientific). The flow rate was set at 200 nl/min. Buffer A and B were 3% ACN (0.1% FA) and 90% ACN (0.1% FA), respectively. The peptides were separated with a 7–30% B gradient in 118 min. Peptides were eluted from the column and nanosprayed directly into the mass spectrometer. The mass spectrometer was operated in a data-dependent mode. Parameters were set as followed: MS1 resolution 60,000, AGC target 4e5, maximum IT 50 ms, scan range 350 to 2000 m/z, dynamic exclusion 45 s, charge inclusion 2–6, cycle time 2 s; MS2 resolution 15,000, AGC target 2e5, maximum IT 105 ms, and an NCE of 35.

TMT-labeled deglycosylated peptides of PDAC as well as CHO cells were analyzed on an Orbitrap Fusion Lumos mass spectrometer (Thermo Scientific). The peptides were separated on an in-house packed C18 column (75 μm × 28 cm, (1.9 μm Reprosil-Pur C18-AQ beads (Dr. Maisch GmbH)) lined up with an EASY-nLC 1200 (Thermo Fisher Scientific). The flow rate was set at 200 nl/min. Buffer A and B were 3% ACN (0.1% FA) and 90% ACN (0.1% FA), respectively. The peptides were separated with a 6–30% B gradient in 84 min. Peptides were eluted from the column and nanosprayed directly into the mass spectrometer. The mass spectrometer was operated in a data-dependent mode. Parameters were set as followed: MS1 resolution 60,000, AGC target 4e5, maximum IT 50 ms, scan range 350 to 1800 m/z, dynamic exclusion 45 s, charge inclusion 2–6, cycle time 2 s; MS2 resolution 50,000, AGC target 2e5, maximum IT 105 ms, and an NCE of 37.

### Data analysis of label-free and TMT data sets

The raw files of label-free samples were searched using SEQUEST in Proteome Discoverer (version 1.4, Thermo Fisher Scientific) against the UniProt/Swiss-Prot human protein database (released in April 2019) for HCC tumor and normal samples, a UniProt CHO protein database (released February 2016) for the samples of CHO cells, and a UniProt bovine protein database for the samples of fetuin and RNase B with the following constraints: tryptic peptides with a maximum of two missed cleavages were allowed; MS1 and MS2 tolerances were set at 10 ppm and 0.06 Da, respectively; Carbamidomethylation (C, +57.021 Da) was set as static modification; oxidation (M, +15.999 Da), deamidation (N, +0.984 Da), HexNAc (N, +203.079 Da), and HexNAc(1)dHex(1) (N, +349.137 Da) were selected as dynamic modifications. The search results were evaluated at a false discovery rate (FDR) < 1% and a requirement of ≥2 peptides per protein. The raw files of TMT-labeled HCC tumor and normal samples were searched against the same human protein database via MS-GF + v2016.02.12 with similar search constraints except precursor mass tolerance was set at 20 ppm, TMT of lysine and N-terminus (+229.162932 Da) were additionally selected as dynamic modifications. The raw files of TMT-labeled PDAC tumor and normal samples were searched against a RefSeq human protein database (released June 2018), while the raw files of TMT-labeled CHO samples were searched against the aforementioned CHO protein database. The search results were filtered by peptide-matched spectrum (PSM)-level FDR < 1% with ≥1 PSM per peptide and ≥1 peptide per protein.

CHO and RNase B glycoproteomic raw data files were converted to universal format mzML files using the msconvert tool 3.0 from ProteoWizard, and searched with the GPQuest search engine (version 2.1) with the following modifications: static carbamidomethylation (C, +57.021464 Da) and dynamic oxidation (M, +15.9949 Da). GPQuest was applied to identify intact N-linked glycopeptides to MS/MS spectra using two approaches: searching spectra containing oxonium ions (“oxo-spectra”) and identifying intact N-linked glycopeptides. The oxonium ions were used as the signature features of the glycopeptides from the MS/MS spectra, which were caused by the fragmentation of glycans attached to intact glycopeptides in the mass spectrometer. The MS/MS spectra containing the oxonium ions (m/z 204.0966) in the top 10 abundant peaks were considered as the potential glycopeptide candidates. The CHO and RNase B intact N-linked glycopeptides were identified by using GPQuest to search against custom glycopeptide and glycan databases specific to the CHO and RNase B data types. Each tandem mass spectrum was first processed in a series of preprocessing procedures, including spectrum de-noising, intensity square root transformation^65^, oxonium ion evaluation, and glycan type prediction^66^. The top 100 peaks in each preprocessed spectrum were matched to the fragment ion index generated from a peptide sequence database to identify all the candidate peptides. All the qualified (≥6 fragment ions matchings) candidate peptides were compared with the spectrum again to calculate the Morpheus scores^67^ by considering all the peptide fragments, glycopeptide fragments, and their isotope peaks. The peptide having the highest Morpheus score was then assigned to the spectrum. The mass gap between the assigned peptide and the precursor mass was searched in the glycan database to find the associated glycan. The best hits of all “oxo-spectra” were filtered by precursor isotopes distribution fitting score and then ranked by the Morpheus score in descending order, in which those with FDR < 1% and covering >10% total intensity of each tandem spectrum were reserved as qualified identifications. The precursor mass tolerance was set as 10 ppm, and the fragment mass tolerance was 20 ppm. The search results were then filtered by PSM-level FDR < 1% with ≥1 PSM per peptide and ≥1 peptides per protein, which controlled the final N-glycopeptide-level FDR to <1%.

The raw files of time-course experiments were searched against the aforementioned CHO protein database and quantified via MaxQuant 1.6.17 using the following settings: Carbomidomethylation (C, +57.021 Da) was set as static modification; HexNAc (N, +203.079 Da), and HexNAc(1)dHex(1) (N, +349.137 Da) were manually added into MaxQuant and set as variable modifications along with oxidation (M, +15.999 Da); LFQ was selected for label-free quantification; peptide tolerance was set as 10 ppm and MS/MS match tolerance was set as 0.06 Da; FDR < 1% for PSM- and protein-levels.

The identified N-linked glycosites were considered as positive results if and only if the asparagine (N) residues occurred at the consensus motif of N-X-S/T, where X can be any amino acid residue except proline. A PSM mapped to a partially deglycosylated peptide (i.e., CF N-acetylglucosamine or N-acetylglucosamine) was included in final results only if at least one of the oxonium ions at m/z 126.055, m/z 138.055, m/z 186.066, or m/z 204.087 was detected, in line with the criteria used for identification of the CF site in the previous study^28^. Of note, glycopeptides with multiple glycosylation sites that were simultaneously modified by HexNAc-Fuc and HexNAc were excluded from the final results due to the potential loss of core fucose during MS/MS analysis^27–29,36,68,69^.

For TMT data, each TMT channel was normalized against the reference channel and then log-transformed and median normalized across all channels. Differential analysis of HCC data was conducted by computing median log2 fold change and using a two-sided *t* test with *p*-value adjusted (FDR) via Benjamini–Hochberg method. Differential analysis of PDAC data was performed by calculating median log2 fold change and conducting paired two-sided *t* test. KEGG pathway enrichment analysis of the proteins from the upregulated CF glycosylation events of HCC was carried out using Over-Representation Analysis in *WebGestalt*^40^ with hypergeometric test and multiple test adjustment via Benjamini–Hochberg method.

### Reporting summary

Further information on research design is available in the Nature Research Reporting Summary linked to this article.

## Supplementary information

Supplementary Information

Description of Additional Supplementary Information

Supplementary Data 1

Supplementary Data 2

Supplementary Data 3

Supplementary Data 4

Supplementary Data 5

Supplementary Data 6

Supplementary Data 7

Supplementary Data 8

Supplementary Data 9

Supplementary Data 10

Supplementary Data 11

Supplementary Data 12

Supplementary Data 13

Supplementary Data 14

Reporting Summary

## Data Availability

All MS data that support the finding of this study are publicly available in MassIVE under massive.ucsd.edu with project identifier MSV000086576 [https://doi.org/doi:10.25345/C53V2T]. Source data are provided in this paper.

## Source data

Source Data
