# Supplementary material for: Characterization of core fucosylation via sequential enzymatic treatments of intact glycopeptides and mass spectrometry analysis
Source: Nat Commun. 2022 Jul 7;13:3910. doi: 10.1038/s41467-022-31472-4 (PMC9262967; doi:10.1038/s41467-022-31472-4)
Supplement: Supplementary file 1 — Supplementary Information [file 41467_2022_31472_MOESM1_ESM.pdf]

Characterization of core fucosylation via sequential enzymatic treatments of intact glycopeptides and mass spectrometry analysis

Liwei Cao[1,#], T. Mamie Lih[1,#], Yingwei Hu[1], Michael Schnaubelt[1], Shao-Yung Chen[1], Yangying Zhou[1], Chuanyu Guo[2], Mingming Dong[1], Weiming Yang[1], Rodrigo Vargas Eguez[1], Lijun Chen[1], David J. Clark [1], Akrit Sodhi[2], Qing Kay Li[1], Hui Zhang[1,*]

[1] Department of Pathology, Johns Hopkins University, Baltimore, Maryland 21231, United States

[2] Wilmer Eye Institute, Johns Hopkins University School of Medicine, Baltimore, Maryland 21287 United States

[#] These authors contributed equally to this work

*Correspondence to: huizhang@jhu.edu

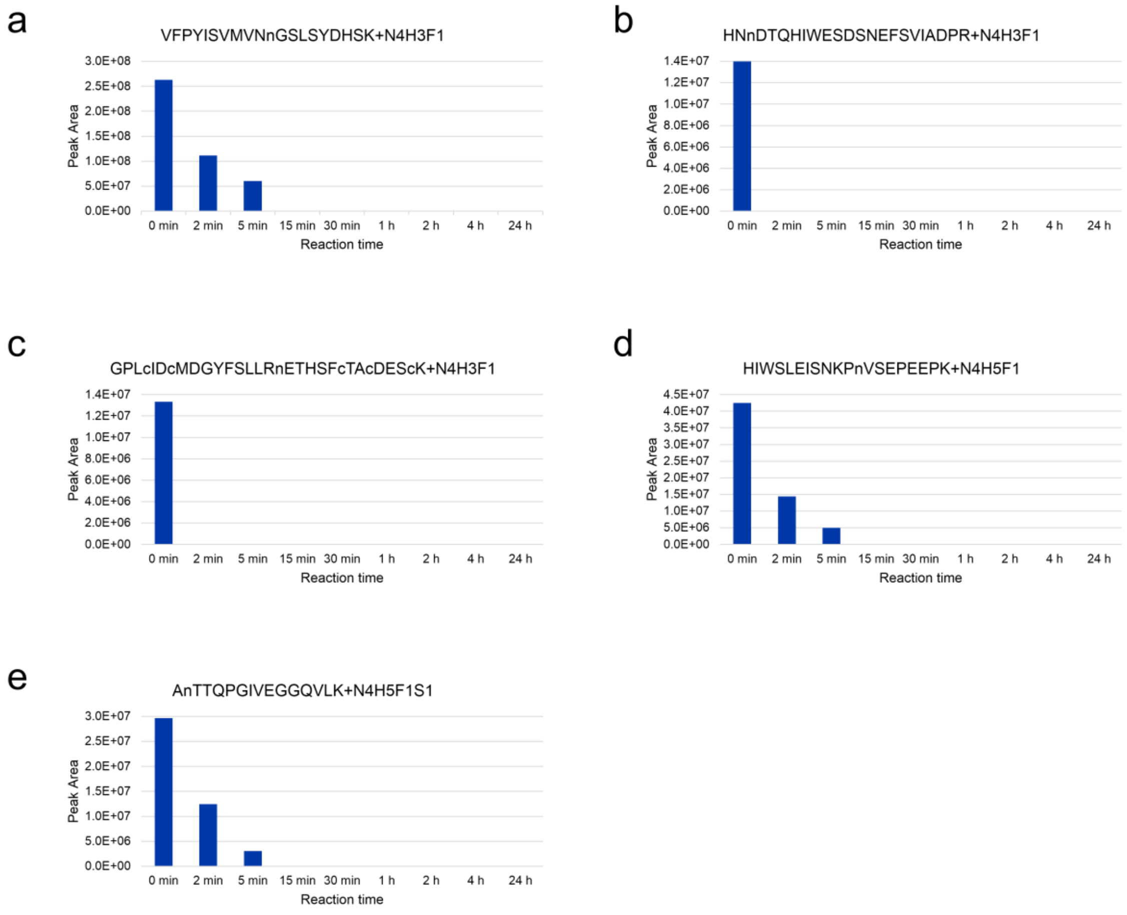

Supplementary Figure 1. Assessment of the completeness of the enzymatic reaction using Endo F3 on CHO cells. a) The peak area of the CF biantennary glycopeptide (VFPYISVMVNnGSLSYDHSK-N4H3F1, Uniprot ID: G3H177) from LMAN2 at different time points. **b)** The peak area of the CF biantennary glycopeptide (HNnDTQHIWESDSNEFSVIADPR-N4H3F1, Uniprot ID: G3HQM6) from endoplasmin at different time points. **c)** The peak area of the CF biantennary glycopeptide (GPLcIDcMDGYFSLLRnETHSFcTAcDEScK-N4H3F1, Uniprot ID: G3IC16) from protein disulfide-isomerase at different time points. **d)** The peak area of the CF biantennary glycopeptide (HIWSLEISNKPnVSEPEEPK-N4H5F1, Uniprot ID: G3HR95) from carboxypeptidase D at different time points. **e)** The peak area of the CF biantennary glycopeptide (AnTTQPGIVEGGQVLK-N4H5F1S1, Uniprot ID: G3HHR9) from integrin alpha-V at different time points. All detectable intact CF biantennary glycopeptides in the

glycopeptide fraction of the CHO digest were listed in Supplementary Figure 1 and Figure 2. Core fucosylation was evidenced by the ion consisting of the peptide and the glycan moiety (HexNAc(1)dHex(1)). c: carbomidomethylation of cysteine; n: glycosylated site; N: HexNAc; H: Hex; F: fucose; S: sialic acid.

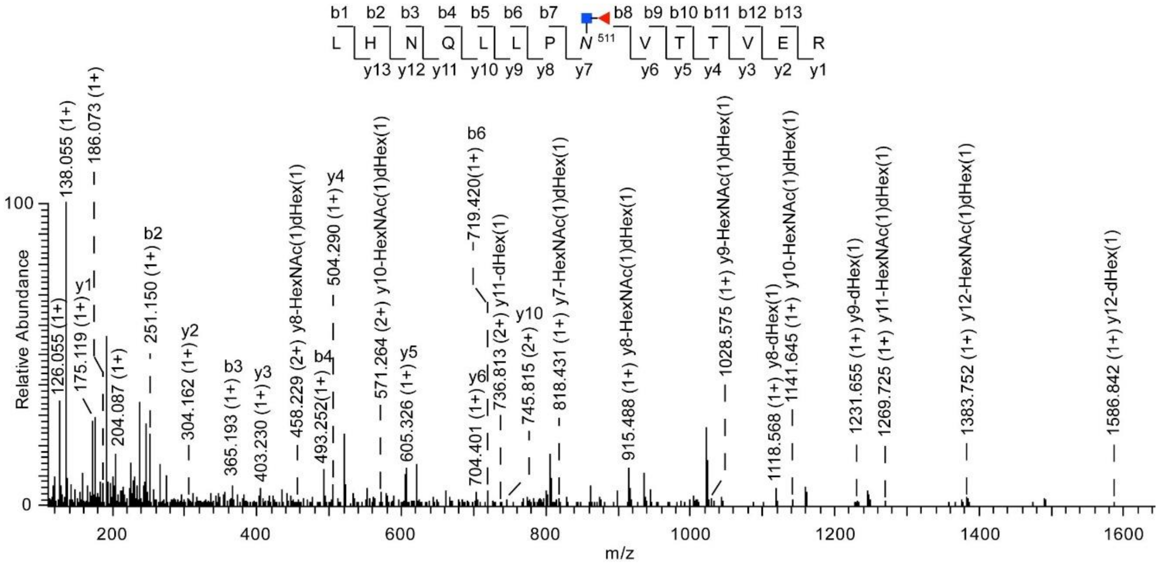

Supplementary Figure 2. MS/MS spectrum of a glycopeptide with CF glycan. MS/MS spectrum of a peptide, LHNQLLP*N*^{511}VTTVER from glutathione hydrolase 1 proenzyme (GGT1, UniProt ID: P19440). The site (*N*^{511}) was core fucosylated (attach to HexNAc-Fuc, Endo F3 treated). Keys: b-HexNAc(1)dHex(1) represents a disaccharide, HexNAc(1)dHex(1), is lost from this b ion; y-HexNAc(1)dHex(1) represents a disaccharide, HexNAc(1)dHex(1), is lost from this y ion; b-dHex(1) represents a monosaccharide residue, dHex(1), is lost from this b ion; y-dHex(1) represents a monosaccharide residue, dHex(1), is lost from this y ion; b-HexNAc(1) represents a monosaccharide residue, HexNAc(1), is lost from this b ion; y-HexNAc(1) represents a monosaccharide residue, HexNAc(1), is lost from this y ion.
