# Supplementary material for: Characterization of core fucosylation via sequential enzymatic treatments of intact glycopeptides and mass spectrometry analysis
Source: Nat Commun. 2022 Jul 7;13:3910. doi: 10.1038/s41467-022-31472-4 (PMC9262967; doi:10.1038/s41467-022-31472-4)
Supplement: Supplementary file 2 — Description of Additional Supplementary Information [file 41467_2022_31472_MOESM2_ESM.pdf]

Description of Additional Supplementary Files

Supplementary Data 1: Reproducibility of the entire STAGE workflow.

Supplementary Data 2: Identification of CF and PNGase F-modified glycosites in Fetuin.

Supplementary Data 3: Number of identified CF glycosites in fetuin (three known N-linked glycosites) using the STAGE method with different amounts of starting material.

Supplementary Data 4: Total glycosylation sites and CF sites (from Endo F3-treated) identified in HCC normal (Normal) and tumor (Tumor) tissues from label-free data.

Supplementary Data 5: The list of CF glycopeptides with multiple glycosites.

Supplementary Data 6: Distribution of CF sites originated from 561 CF site-contained proteins (CF proteins) in label-free data.

Supplementary Data 7: The list of glycopeptides identified via quantitative analysis using STAGE method coupled with TMT labeling for HCC tumor and normal tissues.

Supplementary Data 8: Glycosylation events quantified in at least 75% of the samples (log-transformed and median normalized).

Supplementary Data 9: The list of glycosylation events that were quantified in 75% of TMT reporter channels and showed differential expression between Tumor and Normal tissues (FDR <0.01 and FC ≥ 1.5).

Supplementary Data 10: Differential analysis of core fucosylation between HCC tumor and normal tissues using label-free data.

Supplementary Data 11: KEGG pathways of up-regulated glycoproteins in Tumor and Normal tissues.

Supplementary Data 12: The list of glycopeptides identified via quantitative analysis using STAGE method coupled with TMT labeling for PDAC paired tumor and NAT tissues.

Supplementary Data 13: Glycosylation events quantified in at least 75% of the PDAC samples (log-transformed and median normalized).

Supplementary Data 14: Differential expression of glycopeptides between PDAC tumors and paired NATs.
