# Supplementary material for: Characterization of core fucosylation via sequential enzymatic treatments of intact glycopeptides and mass spectrometry analysis
Source: Nat Commun. 2022 Jul 7;13:3910. doi: 10.1038/s41467-022-31472-4 (PMC9262967; doi:10.1038/s41467-022-31472-4)
Supplement: Supplementary file 17 — Reporting Summary [file 41467_2022_31472_MOESM17_ESM.pdf]

nature portfolio

Corresponding author(s): Hui Zhang

Last updated by author(s): May 20, 2022

Reporting Summary

Nature Portfolio wishes to improve the reproducibility of the work that we publish. This form provides structure for consistency and transparency in reporting. For further information on Nature Portfolio policies, see our Editorial Policies and the Editorial Policy Checklist.

Statistics

For all statistical analyses, confirm that the following items are present in the figure legend, table legend, main text, or Methods section.

n/a	Confirmed	
☐	☒	The exact sample size (n) for each experimental group/condition, given as a discrete number and unit of measurement
☒	☐	A statement on whether measurements were taken from distinct samples or whether the same sample was measured repeatedly
☐	☒	The statistical test(s) used AND whether they are one- or two-sided<br>*Only common tests should be described solely by name; describe more complex techniques in the Methods section.*
☒	☐	A description of all covariates tested
☐	☒	A description of any assumptions or corrections, such as tests of normality and adjustment for multiple comparisons
☒	☐	A full description of the statistical parameters including central tendency (e.g. means) or other basic estimates (e.g. regression coefficient) AND variation (e.g. standard deviation) or associated estimates of uncertainty (e.g. confidence intervals)
☐	☒	For null hypothesis testing, the test statistic (e.g. F, t, r) with confidence intervals, effect sizes, degrees of freedom and P value noted<br>*Give P values as exact values whenever suitable.*
☒	☐	For Bayesian analysis, information on the choice of priors and Markov chain Monte Carlo settings
☒	☐	For hierarchical and complex designs, identification of the appropriate level for tests and full reporting of outcomes
☒	☐	Estimates of effect sizes (e.g. Cohen's d, Pearson's r), indicating how they were calculated

Our web collection on statistics for biologists contains articles on many of the points above.

Software and code

Policy information about availability of computer code

Data collection	Commercial software Thermo Scientific Xcalibur 4.3 is employed to acquire mass spectrometry raw files.
Data analysis	Commercial software SEQUEST in Proteome Discoverer 1.4 and freely available software (MS-GF+ v2016.02.12, MaxQuant 1.6.17, GPQuest 2.1, Msconvert 3.0) are employed to analyze proteomic data present in this study.

For manuscripts utilizing custom algorithms or software that are central to the research but not yet described in published literature, software must be made available to editors and reviewers. We strongly encourage code deposition in a community repository (e.g. GitHub). See the Nature Portfolio guidelines for submitting code & software for further information.

Data

Policy information about availability of data

All manuscripts must include a data availability statement. This statement should provide the following information, where applicable:

- Accession codes, unique identifiers, or web links for publicly available datasets
- A description of any restrictions on data availability
- For clinical datasets or third party data, please ensure that the statement adheres to our policy

All MS data that support the finding of this study are publicly available in MassIVE under massive.ucsd.edu with project identifier MSV000086576 (https://doi.org/doi:10.25345/C53V2T). Source data are provided as a Source Data file.

Field-specific reporting

Please select the one below that is the best fit for your research. If you are not sure, read the appropriate sections before making your selection.

☒ Life sciences ☐ Behavioural & social sciences ☐ Ecological, evolutionary & environmental sciences

For a reference copy of the document with all sections, see nature.com/documents/nr-reporting-summary-flat.pdf

Life sciences study design

All studies must disclose on these points even when the disclosure is negative.

Sample size	No sample size calculation was performed since the manuscript is about the method development instead of a clinical study.
Data exclusions	No data were excluded from the analyses
Replication	Unlabeled deglycosylated peptides of CHO cells were prepared in three technical replicates for evaluation of reproducibility, and each sample was analyzed on mass spectrometer once. TMT labeled peptides were fractionated into 12 fractions, and each fraction was run on mass spectrometer once. All attempts at replication were successful.
Randomization	Randomization was not applicable to the study since the manuscript is about the method development instead of a clinical study. The samples used in this study were to ensure that the developed method was applicable to complex biological samples.
Blinding	Blinding was not applicable to the study since the manuscript is about the method development instead of a clinical study.

Reporting for specific materials, systems and methods

We require information from authors about some types of materials, experimental systems and methods used in many studies. Here, indicate whether each material, system or method listed is relevant to your study. If you are not sure if a list item applies to your research, read the appropriate section before selecting a response.

Materials & experimental systems

n/a	Involved in the study	
☒	☐	Antibodies
☐	☒	Eukaryotic cell lines
☒	☐	Palaeontology and archaeology
☒	☐	Animals and other organisms
☐	☒	Human research participants
☒	☐	Clinical data
☒	☐	Dual use research of concern

Methods

n/a	Involved in the study	
☒	☐	ChIP-seq
☒	☐	Flow cytometry
☒	☐	MRI-based neuroimaging

Eukaryotic cell lines

Policy information about cell lines

Cell line source(s)	The CHO cell line was a gift from Dr. Michael J Betenbaugh (Johns Hopkins University).
Authentication	The CHO cell line used was not authenticated.
Mycoplasma contamination	The CHO cell line used was not tested for mycoplasma contamination.
Commonly misidentified lines (See ICLAC register)	The CHO cell line was not a commonly misidentified cell line according to International Cell Line Authentication Committee.

Human research participants

Policy information about studies involving human research participants

Population characteristics	Patients with HCC (2 males, 2 females between the age group of 36-70) or PDAC (4 males, 2 females between the age group of 42-75) were collected.
Recruitment	Four HCC tumors and four normal liver tissues were included, composed of two pairs of tumor and tumor-matched NATs, two NATs without paired tumor tissues, and two tumor tissues without paired normal tissues. The specimens were collected from surgically resected liver samples. All specimens were snap-frozen and stored at -80°C until use. The hematoxylin and eosin (H&E) stained sections were reviewed by an American Pathology Board certified pathologist (Q.K.L.) to ensure the representation of tumor and normal area. Similarly, in the study of PDAC, 6 PDAC tumors and paired NATs were carefully evaluated histologically and processed according to Clinical Proteomic Tumor Analysis Consortium (CPTAC) guidelines.
Ethics oversight	HCC samples were obtained from The Johns Hopkins Hospital (JHH). Four HCC tumors and 4 normal liver tissues as well as 6 PDAC tumors and 6 paired PDAC NATs were carefully evaluated histologically and included in this study. The donors have given informed consent for their samples being used for research. All human specimens were existing specimens and subjects cannot be identified. The study was exempted for human subject research based on Category 4 - Secondary Research. Participants did not receive financial compensation.

Note that full information on the approval of the study protocol must also be provided in the manuscript.
